# Waist Circumference Is an Anthropometric Parameter That Identifies Women with Metabolically Unhealthy Phenotypes

**DOI:** 10.3390/nu10040447

**Published:** 2018-04-04

**Authors:** Nathaly Torres-Castillo, Wendy Campos-Perez, Karina Gonzalez-Becerra, Iván Hernandez-Cañaveral, Barbara Vizmanos, José Muñoz-Valle, Erika Martinez-Lopez

**Affiliations:** 1 Service of Molecular Biology in Medicine, Civil Hospital Fray Antonio Alcalde, 44280 Guadalajara, Mexico; nathaly_10@hotmail.com (N.T.-C.); wendy_yareni91@hotmail.com (W.C.-P.); kariglezb@gmail.com (K.G.-B.); 2 Department of Molecular Biology and Genomic, Health Sciences University Center, University of Guadalajara, 44340 Guadalajara, Mexico; 3 Department of Microbiology and Parasitology, Health Sciences University Center, University of Guadalajara, 44340 Guadalajara, Mexico; ivanhzc21@yahoo.com.mx; 4 Department of Human Reproduction, Health Sciences University Center, University of Guadalajara, 44340 Guadalajara, Mexico; bvizmanos@yahoo.com.mx; 5 Institute of Research in Biomedical Sciences, University of Guadalajara. 44340 Guadalajara, Mexico; biologiamolecular@hotmail.com

**Keywords:** metabolically healthy phenotype, prevalence, anthropometric parameters, adiponectin

## Abstract

Metabolically healthy (MH) and metabolically unhealthy (MUH) phenotypes can be present in any subject independently of their body mass index (BMI). However, factors related to the presence of these phenotypes are poorly understood. Therefore, the aim of this cross-sectional study is to describe the prevalence and characteristics associated with the MH and MUH phenotypes in Mexican subjects with different BMI categories. Anthropometric and biochemical parameters were evaluated after 12 h of fasting. HMW (High Molecular Weight) adiponectin and insulin levels were measured by ELISA (enzyme-linked immunosorbent assay). A total of 345 subjects were included, of which, 73.9% were women. The prevalence of the MH phenotype was 69.9%, 46.7%, and 19% in normal weight, overweight, and obesity, respectively. ROC (receiver operating characteristic) curve analysis showed that the waist circumference demonstrated a statistical significance (*p* < 0.01) in detecting the MUH phenotype in each BMI group only in women. Furthermore, subjects with lower HMW adiponectin levels showed a 2.1 increased risk of presenting the MUH phenotype. In conclusion, in this Mexican population, waist circumference was an anthropometric parameter that identified women with the MUH phenotype in all BMI categories and hypoadiponectinemia was a risk factor for the presence of this phenotype.

## 1. Introduction

Currently, overweight and obesity are defined as an abnormal or excessive accumulation of fat that may impair health [1]. The worldwide prevalence of obesity has doubled between 1980 and 2014 [1]. In Mexico, more than 70% of adults are overweight or obese [2]. Regardless of obesity being widely linked to several metabolic alterations and chronic diseases like dyslipidemia, insulin resistance, cardiovascular diseases, and type 2 diabetes mellitus (T2DM), not every obese subject presents these complications [3].

Unexpectedly, a body mass index (BMI) > 30 kg/m^2^ does not necessarily lead to metabolic disorders; in fact, some individuals with obesity according to their BMI, may have better metabolic profiles than expected [4]. These subjects are known as “metabolically healthy obese” [3,4] because they can be characterized by insulin sensitivity, lipid profile, blood pressure, and inflammation markers within the normal range, despite an elevated fat mass [3,4]. However, in obese subjects, the absence of risk factor values exceeding the metabolic syndrome definition threshold should not be equated to being “healthy”. 

On the contrary, subjects with normal weight can have an unhealthy metabolic profile known as metabolically unhealthy (MUH) phenotype that may be associated with a higher risk of chronic diseases. This suggests that despite a normal BMI in an individual, being MUH could increase the risk of chronic diseases and its comorbidities [5,6]. 

Prevalence of the metabolically healthy (MH) phenotype in obese individuals can vary from 10% to almost 50% depending of the diagnostic criteria [7]; however, most authors consider metabolic syndrome parameters and establishing insulin sensitivity to define this MH phenotype [7,8,9,10]. Furthermore, other characteristics that could identify subjects with the MUH phenotype, are not clearly understood. 

Although BMI is commonly used to identify overweight and obesity subjects, it does not provide enough information about the amount of body fat and even less about metabolic alterations, which makes it an unsuitable parameter to identify subjects with the MUH phenotype. On the other hand, waist circumference has been recognized as an anthropometric parameter to predict cardio-metabolic alterations, and is even more representative than total body fat, and therefore, it could be a useful tool to differentiate individuals with the MUH phenotype [11,12]. 

Moreover, Scott Ahl et al. reported that in North American white population, waist circumference was inversely correlated with plasma adiponectin serum levels, which in a low quantity was associated with the MUH phenotype, however, these observations must be confirmed in other populations [13].

In the Mexican population, the prevalence and characteristics related to the MH and MUH phenotypes have been scarcely studied. Therefore, the aim of this study is to describe the prevalence and characteristics associated with these two phenotypes in Mexican subjects classified in different BMI categories.

## 2. Materials and Methods

### 2.1. Study Population

**Figure 1:**
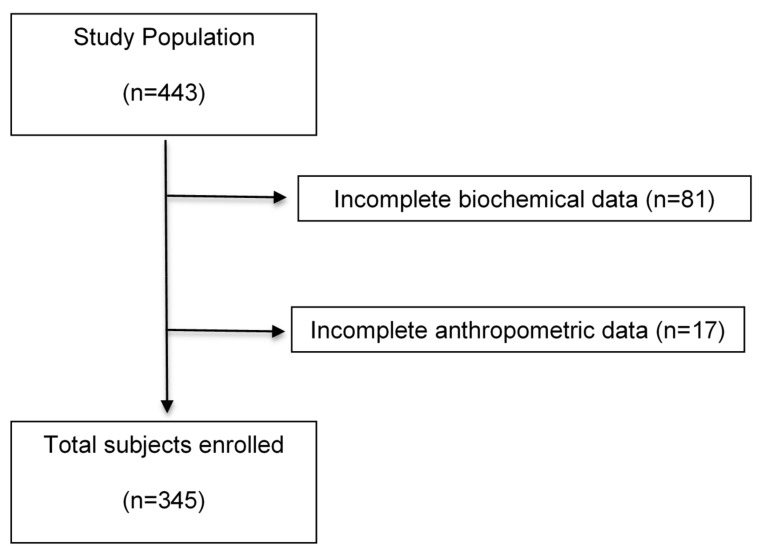
Flow diagram of subjects included in this study.

In this cross-sectional study, a total of 443 unrelated adults from Western Mexico were recruited. However, only 345 had their complete data and therefore, were included (Figure 1). All individuals were recruited via posters and brochures from December 2011 to May 2012. The study was conducted in the Medical Molecular Biology Service of the “Fray Antonio Alcalde” Civil Hospital of Guadalajara, Jalisco, Mexico. Inclusion criteria included mestizos from Western Mexico, aged 18–65 years old, with a BMI ≥ 18.5 kg/m^2^ and classified as normal weight, overweight, or obesity according to the BMI. Participants provided a written informed consent before their inclusion in the study. Subjects were excluded if they had any prescribed medication for chronic diseases, such as T2DM, cardiovascular, liver, kidney, and the pancreas, since such medication could alter blood pressure, serum glucose, and/or lipid profile and thus, the ability to detect MH subjects could be skewed. Participants were also excluded if they were drug addicts, smokers or drinkers (alcohol intake > 20 g/day and > 40 g/day in women and men, respectively [14]. Women who were pregnant or breastfeeding were excluded as well. This study was approved by the Ethics Committee for Human Research of the University of Guadalajara (Register number: CI/019/2010) and all procedures were performed according to the Declaration of Helsinki (2013).

### 2.2. Definition of the Metabolically Healthy Phenotype (MH) and Metabolically Unhealthy Phenotype (MUH)

There is no a universal definition for the MH phenotype, however, most authors [7,8,9,10,15] use the cut-off points for metabolic syndrome by the NCEP-ATP III (National Cholesterol Education Program Adult Treatment Panel III) [16] and insulin resistance, to identify subjects with this phenotype. Therefore, in this study, for each BMI category, the following criteria were considered: blood pressure ≥ 130/85 mmHg, triglycerides ≥ 150 mg/dL, HDL (high-density lipoprotein)-cholesterol < 40 mg/dL in men and < 50 mg/dL in women, fasting glucose ≥ 100 mg/dL and the homeostasis model assessment of insulin resistance (HOMA-IR) > 2.5 [17]. If subjects had one or none of these altered cut-off points, they were considered as MH phenotype; otherwise, they were classified as MUH phenotype (≥2 criteria). 

### 2.3. Anthropometric Measurements

Anthropometric parameters were measured after 12 h of fasting. Measurements were performed with light clothes and without shoes. Tetrapolar electrical bioimpedance was utilized to determine body composition including protein mass, fat mass and body fat percentage (InBody 3.0, Biospace Co., Seoul, Korea). BMI was calculated dividing weight in kilograms by height in meters squared (kg/m^2^). Height measurement was determined using a stadiometer with a precision of 1 mm (Rochester Clinical Research, Inc., New York, NY, USA). Waist circumference was measured in the narrowest diameter between the last rib and the iliac crest, and hip circumference was determined at the maximum posterior protuberance level of the gluteus using a Lufkin Executive® Thinline 2 mm measuring tape (Lufkin Executive Thinline, W606PM, MD, USA). Blood pressure was evaluated with a LifeSource digital sphygmomanometer (LifeSource, Milpitas, CA, USA), after a period of at least 15 min rest. Subjects were instructed to sit in the chair with their backs touching the back of the chair and with their arm resting on a horizontal surface and with legs not crossing. Two blood pressure measurements were made, and the average was registered. 

### 2.4. Biochemical Analysis

Venous blood was taken after an overnight fast. Immediately, serum was separated and analyzed. Measurements of glucose, triglycerides, total cholesterol, high-density lipoprotein cholesterol (HDL-c), and conventional C-reactive protein (CRP) were performed by dry chemistry using a Vitros 250 Analyzer (Ortho Clinical Diagnostics, Johnson and Johnson, Co., Rochester, NY, USA). Low-density lipoprotein cholesterol (LDL-c) was calculated using the Friedewald formula [18] excluding subjects with serum triglyceride values above 400 mg/dL [19]. 

Very-low-density lipoprotein cholesterol (VLDL-c) was calculated as total cholesterol minus (LDL-c + HDL-c). For quality control purposes, a human pooled serum and a commercial control serum was used to count for accuracy of the biochemical measurements. High molecular weight isoform (HMW) adiponectin and insulin levels were measured by an ELISA assay according to the manufacturer’s recommendations (ALPCO Diagnostics, Salem, NH, USA, and Monobind Inc., Lake Forest, CA, USA, respectively). Hypoadiponectinemia was defined as adiponectin levels < 3 μg/mL [20]. The HOMA-IR was calculated according to the model proposed by Matthews [17]. 

### 2.5. Statistical Analysis

Values are expressed as mean ± standard deviation (SD) or percentage as appropriate. All quantitative variables were assessed for normal distribution with the Kolmogorov–Smirnov test. Comparisons between two groups were analyzed with the Student-*T* test or Mann–Whitney *U* test. The comparisons between the three MH phenotype groups were done with ANOVA (analysis of variance) or Kruskal–Wallis test, as appropriate. ROC curve analysis was performed to evaluate if anthropometric variables were predictors of the MUH phenotype based on the definition previously described. Binary logistic regression analysis was performed to identify risk factors associated with the presence of the MUH phenotype. A *p* value < 0.05 was considered statistically significant. All statistical analyses were performed using SPSS v. 20.0 software (IBM Corp., Armonk, NY, USA). 

## 3. Results

### 3.1. Prevalence of MH Phenotype

A total of 345 subjects were recruited to participate in this cross-sectional study, of which, 73.9% were women. Based on BMI categories, 132 (38.3%) subjects were classified as normal weight, 108 (31.3%) overweight, and 105 (30.4%) had obesity. The prevalence of the MH phenotype in each BMI category was 69.9% for normal weight, 46.7% for overweight and 19.0% for obesity. 

### 3.2. Demographic, Anthropometric, and Clinical Characteristics in MH and MUH Phenotypes

Comparisons of demographic, anthropometric, and clinical variables between MH phenotype vs. MUH phenotype demonstrated that weight and waist circumference were lower in the MH phenotype in each BMI group. Protein mass was lower in the MH than in the MUH phenotype, but only in overweight individuals. Furthermore, comparisons between the three metabolically healthy groups showed that as subjects had a higher BMI, the values of the anthropometric measurements were also increased. These and other significant anthropometric differences are shown in Table 1.

### 3.3. Biochemical Characteristics in MH and MUH Phenotypes

Analysis of biochemical variables indicated that subjects with the MH phenotype had lower levels of triglycerides, HDL-c, glucose, insulin, and HOMA-IR, than its MUH counterpart in each BMI category; nevertheless, these variables were considered to define the MH and MUH phenotypes and such differences were expected. On the other hand, total cholesterol was higher in subjects with the MUH normal weight phenotype than in subjects with the MH normal weight phenotype. Adiponectin levels were higher in the MH than in the MUH phenotype, but only in overweight subjects, however, the three MH groups had the same adiponectin levels. Besides, in the three MH phenotypes, CRP increased as the BMI increased. These results are also presented in Table 1.

### 3.4. ROC Curve to Predict the MUH Phenotype Based on Anthropometric Measurements

Based on the MUH definition used in this study, biochemical and anthropometric variables were analyzed to predict the MUH phenotype, but only waist circumference demonstrated a statistical significance (*p* < 0.01). Because waist circumference displays sexual dimorphism, this analysis was done in males and females, separately. ROC curve analysis in women showed that the cut-off points of waist circumference to detect the MUH phenotype were 73.8 cm in normal weight, 87.3 cm in overweight, and 99.3 cm in subjects with obesity. These results are presented in Table 2. In males, no significant differences were observed (Table 2).

### 3.5. Risk Factors Associated with the MUH Phenotype

It was demonstrated that age was a risk factor associated with the MUH phenotype; in contrast, female gender is a protective factor. Moreover, the protein mass:fat mass ratio was inversely associated with the MUH phenotype, and subjects with hypoadiponectinemia had a 2.106 fold increased risk to present this unhealthy phenotype. These results are shown in Table 3.

## 4. Discussion

The prevalence and characteristics of the MH and MUH phenotypes have been rarely investigated in the Mexican population. In this study, we evaluated both MH and MUH phenotypes in Mexican subjects classified in different BMI categories. The prevalence of the MH phenotype was similar to that reported by Wildman et al., who used similar criteria to define this phenotype. They found a prevalence of 67.2%, 43.6%, and 33.8% in subjects with normal-weight, overweight, and obesity, respectively [21]. Interestingly, in both studies, the prevalence of the MUH phenotype in the normal-weight group was more than 30%, which suggests that even with a normal BMI, almost a third of subjects with normal weight may have higher chances of developing complications associated with obesity, such as T2DM or cardiovascular diseases. The MH phenotype in subjects with obesity has been previously investigated in Mexican population. Aguilar-Salinas et al. reported a prevalence of 36.4% of the MH phenotype in subjects with obesity [9], which was higher than the prevalence found in this study; nevertheless, their criteria to define the MH phenotype only included fasting glucose levels < 126 mg/dL, blood pressure < 140/90 mmHg, and HDL-c levels > 40 mg/dL, contrary to the stricter parameters used in this study. This result also highlights the importance of establishing universal criteria to define these phenotypes.

Since the determinants of the MH phenotype are not clearly understood, we wanted to know the characteristics that distinguish the MH and MUH phenotypes. It was found that in subjects with obesity, those with the MH phenotype had lower BMI than individuals with the MUH phenotype. This finding can be explained as a result of the higher proportion of class II and III obesity subjects; nevertheless, in both phenotypes, most of the subjects had class I obesity. It has also been reported that subjects with obesity class II and III have a worse metabolic profile [22,23]. Interestingly, in this study, despite a higher BMI in the MUH phenotype, no differences were obtained in fat mass and body fat percentage. 

Furthermore, we found that MH subjects had a lower waist circumference compared with their MUH counterparts, even when both had the same body fat percentage. These results are supported by other studies where waist circumference is different between the healthy and unhealthy groups, but there were no differences in body fat percentage [24,25,26,27]. This suggests that not only the amount of body fat is an important factor in the development of metabolic complications, but also, the way fat is distributed. 

In this sense, it has been hypothesized that regardless of the amount of adipose tissue, adipocytes have a limited ability to store lipids, which is different from one person and another [28]. In fact, larger size adipocytes have a lower capacity to store lipids leading to ectopic fat accumulation and metabolic complications [29]. Visceral fat is associated with ectopic fat accumulation [29,30], and it is possible that subjects with a MH phenotype could have more functional adipocytes even when they have equal fat mass compared to MUH subjects. However, because of the cross-sectional design of this study, other studies with an experimental design are necessary to elucidate the mechanism by which these metabolic phenotypes are presented. 

Moreover, ROC curve analysis showed that waist circumference was useful to detect women with an MUH phenotype. To our knowledge, only one study has reported that waist circumference was one of the best anthropometric parameters to predict this phenotype in women [24]. Nevertheless, one of the limitations of this study was the lack of the gold standard methodology to analyze body composition [31], because subjects with the same waist circumference could have different amounts of abdominal visceral fat as previously reported by Harris et al. [32]. Thus, future studies that validate and corroborate waist circumference according to values obtained with the gold standard tools of body composition in this type of population are needed, because this anthropometric measurement could be a useful and cheaper tools in clinical practice to identify subjects with a higher risk of presenting the MUH phenotype.

In this study, age and hypoadiponectinemia were positively associated with the MUH phenotype; in contrast, a higher protein mass:fat mass ratio and female gender were inversely associated with the MUH phenotype. Some studies have found that the prevalence of MUH increases with age [33], and another report found the opposite [34]. In other cases, no differences have been found [35]. However, more studies are needed to clarify these conflicting results. Besides, hypoadiponectinemia was associated with the risk to present the MUH phenotype. In a study of MH and MUH phenotypes in subjects with obesity, Chang C.-S. et al. found that total adiponectin was not a risk factor for the MUH phenotype [25], but they did not measure the HMW isoform. In fact, most of the studies only consider total adiponectin [13,36,37], and not the HMW isoform, which has demonstrated more metabolic effects [38]. Although overweight subjects with the MH phenotype had higher adiponectin levels than the MUH phenotype, the three MH groups presented the same adiponectin parameters, independently of the BMI. Moreover, it has been reported that adiponectin levels have a heritability of up to 50% [39], and the adiponectin gene contains several genetic variants that influence their levels [40], which could in part explain the conflicting results found in different studies. Nevertheless, few studies have focused on these genetic traits in MH and MUH subjects. 

On the other hand, the protein mass:fat mass ratio was inversely associated with the MUH phenotype. To our knowledge, there are no other studies that have analyzed this ratio in both phenotypes, but a higher visceral fat:thigh muscle ratio has been associated with an increased risk of developing metabolic syndrome by six times [41]. Moreover, Kim T.N. et al., found that subjects with the MUH phenotype and a normal weight had an increased risk of low muscle mass than the MH normal weight [42]. 

In agreement with the results found here, a study using the same criteria for the definition of the MH and MUH phenotype showed that in some cases, the female gender was a factor for the MUH phenotype [25]. In addition, Phillips et al., used five different definitions for the MH phenotype, and found that in four of them, women had 2–4 times more likelihood to present the MH phenotype than men [34]. 

The limitations of this study include its sample size, transversal design, and the lack of a gold standard methodology to measure body composition. However, the results observed here, could be a precedent for other studies that include a larger population. In addition, we did not evaluate the physical activity level, which has been considered as a potential factor that affects the metabolic profile and adiponectin levels [43]. Therefore, physical activity should be considered in future investigations since it could be a determinant factor to carry the MH phenotype. 

## 5. Conclusions

In conclusion, waist circumference was an anthropometric parameter that identified women with the MUH phenotype in all BMI categories in this Mexican population. Moreover, hypoadiponectinemia was a risk factor for presenting the MUH phenotype.

## Figures and Tables

**Table 1:** Characteristics in metabolically healthy and metabolically unhealthy phenotypes according to BMI classification.

Variables	MH-NW	MUH-NW	*p*	MH-OW	MUH-OW	*p*	MH-O	MUH-O	*p*	* *p*
Demographic, anthropometric and clinical characteristics										
Sex (M/F)	23/70	12/28	0.527	15/42	22/28	0.067	3/17	15/70	1.000	
Age (year)	32.0 ± 10.7 ^a^	38.3 ± 12.4	0.005	37.7 ± 12.3 ^b^	37.8 ± 10.7	0.966	39.9 ± 13.2 ^b^	39.6 ± 10.8	0.936	0.005
Weight (kg)	59.1 ± 8.4 ^a^	63.5 ± 6.2	0.004	71.1 ± 8.4 ^b^	75.9 ± 8.8	0.006	84.6 ± 9.9 ^c^	93.2 ± 17.2	0.036	<0.001
BMI (kg/m^2^)	21.9 ± 1.9 ^a^	23.1 ± 1.5	<0.001	26.9 ±1.4 ^b^	27.2 ± 1.4	0.254	33.4 ± 2.4 ^c^	36.1 ± 5.4	0.001	<0.001
Protein mass (kg)	11.2 ± 1.8 ^a^	11.5 ± 1.7	0.482	11.8 ± 2.2 ^a,b^	13.0 ± 2.5	0.033	12.4 ± 2.2 ^b^	13.5 ± 2.6	0.096	0.040
Fat mass (kg)	14.5 ± 4.3 ^a^	17.1 ± 3.5	0.001	23.6 ± 6.4 ^b^	23.6 ± 7.8	0.999	34.6 ± 6.6 ^c^	39.5 ± 11.3	0.067	<0.001
BFP (%)	24.5 ± 6.3 ^a^	27.1 ± 5.8	0.026	32.4 ± 6.1 ^b^	30.3 ± 6.6	0.081	41.0 ± 6.7 ^c^	42.1 ± 6.6	0.492	<0.001
PM:FM ratio	0.819 ± 0.342 ^a^	0.720 ± 0.273	0.045	0.544 ± 0.195 ^b^	0.574 ± 0.240	0.382	0.375 ± 0.126 ^c^	0.360 ± 0.102	0.562	<0.001
Waist (cm)	74.8 ± 8.3 ^a^	80.8 ± 7.3	0.000	87.0 ± 7.9 ^b^	91.6 ± 7.1	0.002	100.5 ± 6.9 ^c^	107.7 ± 12.4	0.001	<0.001
Males	83.2 ± 7.7 ^a^	86.3 ± 5.8	0.228	94.5 ± 7.0 ^b^	95.6 ± 5.8	0.595	103.0 ± 11.3 ^c^	114.9 ± 11.9	0.192	<0.001
Females	72.3 ± 6.8 ^a^	77.7 ± 6.0	0.001	84.0 ± 6.3 ^b^	88.6 ± 6.4	0.003	99.6 ± 6.1 ^c^	106.2 ± 11.9	0.003	<0.001
Hip (cm)	95.2 ± 4.8 ^a^	97.5 ± 3.9	0.011	103.7 ± 4.6 ^b^	104.8 ± 4.3	0.199	114.0 ± 6.7 ^c^	121.2 ± 15.0	0.062	<0.001
WHR	0.78 ± 0.08 ^a^	0.83 ± 0.08	0.002	0.84 ± 0.08 ^b^	0.88 ± 0.07	0.019	0.88 ± 0.08 ^b^	0.88 ± 0.07	0.961	<0.001
SBP (mmHg)	108.1 ± 12.6 ^a^	119.1 ± 17.9	<0.001	110.3 ± 11.2 ^a,b^	121.3 ± 16.5	<0.001	116.4 ± 12.0 ^b^	120.7 ± 13.9	0.204	0.021
DBP (mmHg)	68.1 ± 7.4 ^a^	72.6 ± 8.9	0.003	69.7 ± 7.9 ^a^	79.3 ± 11.0	<0.001	75.3 ± 8.7 ^b^	77.9 ± 15.3	0.471	0.001
Biochemical characteristics										
Triglycerides (mg/dL)	93.5 ± 30.1 ^a^	172.8 ± 107.3	<0.001	109.3 ± 40.9 ^b^	205.2 ± 76.7	<0.001	96.3 ± 26.8 ^a,b^	181.9 ± 88.2	<0.001	0.021
Total cholesterol (mg/dL)	174.6 ± 29.8	193.6 ± 41.4	0.012	183.2 ± 39.3	188.7 ± 34.8	0.451	190.4 ± 32.9	193.9 ± 36.1	0.702	0.104
HDL-c (mg/dL)	52.1 ± 12.2 ^a^	40.3 ± 7.7	<0.001	48.0 ± 11.0 ^b^	38.4 ± 12.0	<0.001	46.5 ± 10.7 ^a,b^	38.0 ± 7.1	0.003	0.039
LDL-c (mg/dL)	105.9 ± 27.5 ^a^	115.4 ± 32.8	0.094	114.0 ± 34.2 ^a,b^	109.5 ± 30.6	0.484	125.2 ± 30.6 ^b^	120.2 ± 31.7	0.525	0.026
VLDL-c (mg/dL)	18.7 ± 6.1 ^a^	34.5 ± 21.4	<0.001	21.8 ± 8.2 ^b^	40.4 ± 14.7	<0.001	18.6 ± 5.4 ^a,b^	35.4 ± 15.7	0.000	0.020
Glucose (mg/dL)	83.2 ± 7.2 ^a^	96.6 ± 59.2	0.007	84.2 ± 8.8 ^a,b^	91.3 ± 10.4	<0.001	88.7 ± 8.3 ^b^	98.0 ± 12.5	0.002	0.019
Insulin (μU/mL)	7.4 ± 4.5	13.3 ± 9.9	0.001	7.1 ± 3.6	13.7 ± 8.3	<0.001	8.7 ± 3.7	17.5 ± 8.8	0.000	0.315
HOMA-IR	1.5 ± 1.0	3.0 ± 2.3	<0.001	1.5 ± 0.9	3.1 ± 2.0	<0.001	1.9 ± 0.7	4.3 ± 2.3	0.000	0.272
CRP (mg/L)	4.8 ± 5.0 ^a^	4.7 ± 4.0	0.369	8.0 ± 7.3 ^b^	10.9 ± 11.4	0.259	14.5 ± 9.2 ^c^	12.6 ± 8.5	0.519	<0.001
Adiponectin HMW (μg/mL)	4.3 ± 3.1	3.8 ± 2.6	0.181	3.8 ± 2.0	2.3 ± 1.5	<0.001	3.4 ± 2.3	2.6 ± 1.8	0.124	0.189

MH-NW: Metabolically healthy normal weight, MUH-NW: Metabolically unhealthy normal weight, MH-OW: Metabolically healthy overweight, MUH-OW: Metabolically unhealthy overweight, MH-O: Metabolically healthy obesity, MUH-O: Metabolically unhealthy obesity, M: Male, F: Female, BFP: Body Fat Percentage, PM:FM ratio: Protein mass:fat mass ratio, WHR: Waist to hip ratio, SBP: Systolic Blood Pressure, DBP: Diastolic Blood Pressure, CRP: C-reactive protein, HMW: High Molecular Weight. * *p*: *p* value for comparisons between the three metabolically healthy groups. Means with the same subscript (a/b/c) are not significantly different; means with different subscript (a/b/c) denote a statistically significant difference.

**Table 2:** ROC curve for detecting the metabolically unhealthy phenotype in men and women with normal-weight, overweight, and obesity.

**Variable: Waist Circumference in Men**	**MUH-NW**	**MUH-OW**	**MUH-O**
AUC ± SE	0.647 ± 0.10	0.530 ± 0.10	0.744 ± 0.21
95% CI	0.458–0.835	0.329–0.730	0.325–1.00
*p* value	0.156	0.766	0.193
Sensibility	71.4%	35.7%	66.7%
Specificity	61.5%	73.9%	100%
Cut-off point (cm)	>85.5	>92.5	>99.0
**Variable: Waist Circumference in Women**	**MUH-NW**	**MUH-OW**	**MUH-O**
AUC ± SE	0.738 ± 0.05	0.692 ± 0.06	0.690 ± 0.07
T95% CI	0.633–0.842	0.567–0.816	0.552–0.807
*p* value	<0.001	0.006	0.030
Sensibility	64.8%	75.0%	66.7%
Specificity	76.9%	56.7%	69.0%
Cut-off point (cm)	>73.8	>87.3	>99.3

MUH-NW: Metabolically unhealthy normal weight, MUH-OW: Metabolically unhealthy overweight, MUH-O: Metabolically unhealthy obesity, AUC: Area under the curve, SE: Standard error, CI: Confidence interval.

**Table 3:** Binary logistic regression model for the risk factors associated with the MUH phenotype.

Variable	B	*p*	OR	95% CI
*R*^2^ Nagelkerke = 28.7%				
Sex (M = 0, F = 1)	−1.345	0.006	0.261	0.100–0.680
Age (years)				
18–29		0.011		
30–39	1.167	0.002	3.212	1.533–6.728
40–49	0.745	0.045	2.214	1.019–4.812
≥50	0.911	0.028	2.486	1.101–5.615
PM:FM ratio	−3.556	0.000	0.029	0.006–0.132
Hypoadiponectinemia	0.745	0.017	2.106	1.143–3.880

M: male, F: female, PM:FM ratio: protein mass:fat mass ratio.
